# C3-PRO: Connecting ResearchKit to the Health System Using i2b2 and FHIR

**DOI:** 10.1371/journal.pone.0152722

**Published:** 2016-03-31

**Authors:** Pascal B. Pfiffner, Isaac Pinyol, Marc D. Natter, Kenneth D. Mandl

**Affiliations:** 1 Computational Health Informatics Program, Boston Children's Hospital, Boston, Massachusetts, United States of America; 2 Research Centre for Medical Informatics, University Hospital Zurich, Zürich, Switzerland; 3 Department of Biomedical Informatics, Harvard Medical School, Boston, Massachusetts, United States of America; 4 Department of Pediatrics, Harvard Medical School, Boston, Massachusetts, United States of America; Seoul National University College of Medicine, REPUBLIC OF KOREA

## Abstract

A renewed interest by consumer information technology giants in the healthcare domain is focused on transforming smartphones into personal health data storage devices. With the introduction of the open source *ResearchKit*, Apple provides a framework for researchers to inform and consent research subjects, and to readily collect personal health data and patient reported outcomes (PRO) from distributed populations. However, being research backend agnostic, ResearchKit does not provide data transmission facilities, leaving research apps disconnected from the health system. Personal health data and PROs are of the most value when presented in context along with health system data. Our aim was to build a toolchain that allows easy and secure integration of personal health and PRO data into an open source platform widely adopted across 140 academic medical centers. We present C3-PRO: the *Consent*, *Contact*, *and Community framework for Patient Reported Outcomes*. This open source toolchain connects, in a standards-compliant fashion, any ResearchKit app to the widely-used clinical research infrastructure *Informatics for Integrating Biology and the Bedside* (i2b2). C3-PRO leverages the emerging health data standard *Fast Healthcare Interoperability Resources* (FHIR).

## Introduction

In March 2015, Apple Inc. announced ResearchKit (*http://researchkit.org*), an open source programming framework that begins to commoditize the creation of iPhone research apps. This foray by a consumer information technology (IT) giant into the realm of clinical research follows clear signals by the tech industry of interest in the health IT ecosystem, including release of the Apple *HealthKit*, *Google Fit*, and Samsung *S Health*.

This first iteration of ResearchKit was designed to provide a complete system to conduct a study where patients are recruited directly via a smartphone app. Out of the box functionality included 1) guiding participants through an easily comprehensible consent process, collecting their signed consent on-screen and exporting a PDF, 2) administering surveys for patient-reported outcome (PRO) collection well-optimized for mobile, and 3) collecting personal health data from built-in phone sensors, or devices accessing the phone's HealthKit application programming interface. Five original apps were released alongside the ResearchKit framework, each supported by a recognized medical institution [1,2]. In the meantime, eight official new apps utilizing ResearchKit have become available to the public.

For the researcher to acquire data for storage and analysis, a participant's consent, PRO and device sensor data must be transmitted from the phone. ResearchKit however does not facilitate this transmission step, the original apps used SAGE Bionetworks (*http://sagebase.org*) services.

We extend ResearchKit with C3-PRO: the *Consent*, *Contact*, *and Community framework for Patient Reported Outcomes* (*http://c3-pro.org*). This open source toolchain connects any ResearchKit app to a widely-used clinical research IT infrastructure called *Informatics for Integrating Biology and the Bedside* (i2b2) [3,4], relying on the emerging standard, *Fast Healthcare Interoperability Resources* (FHIR) [5]. Work to adapt our approach to *ResearchStack* (*http://researchstack.org*), the Android analog to ResearchKit currently under development, is underway.

Though ResearchKit will certainly be adapted to diverse trial designs, our first iteration of C3-PRO supports direct enrollment of a completely anonymous cohort with data stored in i2b2. Future iterations will support identified data collection and enable ResearchKit-collected PROs and device data to be concatenated with the electronic health record data stored in i2b2 at scores of academic medical centers.

## Materials and Methods

**Fig 1:**
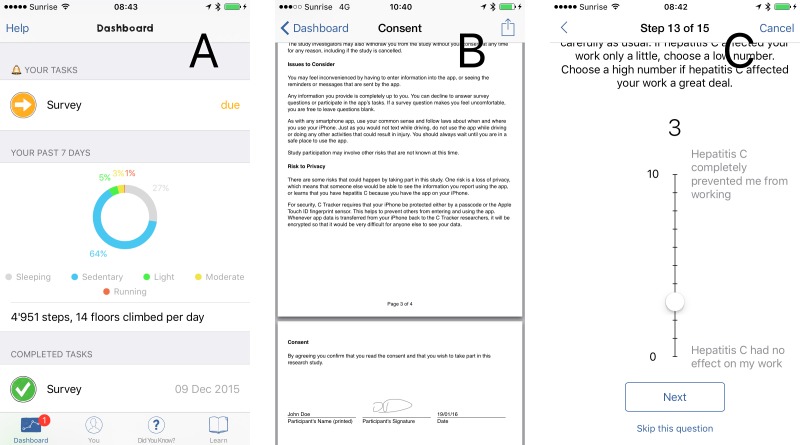
Screenshots of the C Tracker app making use of C3-PRO. **A**) App dashboard for participation overview. **B**) Viewing the generated consent PDF file, including signature and date, on device. **C**) Filling out a short survey, showing one of the questions rendered by ResearchKit.

C3-PRO was created in concert with development of an app, C Tracker [6], to engage patients with hepatitis C in using their smartphones (Fig 1) to report information about themselves that may improve how hepatitis C is treated [7].

The ResearchKit framework, written in Objective-C, serves as the presentation and interaction layer for our technology stack. We use Apple's open source *Swift* programming language (*http://swift.org*) for development of the C3-PRO iOS framework after prior success developing the Swift SMART and FHIR frameworks [8,9].

C3-PRO implements a method for programmatically creating eligibility questions, informed consent sections and participant surveys using FHIR data formats. Participant responses and device-collected activity data and health data stored in HealthKit are likewise represented as FHIR resources when transmitted to the i2b2 backend.

The initial release focuses on anonymous, account-less participation in a study. Therefore, while we use ResearchKit to generate a PDF file of the consent containing the participant's name, signature and date, this file is not transmitted to our backend. Instead, we return an anonymized patient resource, linked to a contract resource representing the consent document, to indicate consent in the research database.

Transmitting PHI over the Internet requires use of strong encryption. We have implemented public-key cryptography on the device so only the research backend is capable of interpreting PHI-containing resources sent from the device.

In order to not publicly expose our research backend we built an interposed C3-PRO “receiver” server that serves as communication point for the app, serving static content to and accepting encrypted data from the smartphone app. This server is written in Java, designed to be scalable and deployed to generic hosting services, currently Amazon Web Services (AWS, *https://aws.amazon.com*). It authenticates requests and stores incoming data to a local queue.

On the research backend, a C3-PRO “consumer” component is continually reading the data stored by the previously described, publicly hosted receiver. It has access to the private decryption key and hence is capable of decrypting incoming data. Decrypted data, in FHIR format, is then sent to a new i2b2 component (“cell”) capable of receiving FHIR resources, our i2b2 FHIR cell. This cell converts data from FHIR format to i2b2's native representation for permanent storage into the i2b2 database.

During development of our codebase, a development environment was set up and improved iteratively, working on app integration, public facing and internal server components simultaneously.

## Results

### Authentication

By design, the phone itself serves as an identifier and authenticator of study participants. The app creates a universally unique identifier (UUID) upon first launch, which is stored in the device's keychain, a cryptographically secured location on iOS devices [10]. Storing the UUID in the device's keychain not only secures it from unauthorized access, it also survives deletion and re-installation of the app. While a participant may withdraw and immediately re-enroll in a trial to submit more data, the same UUID will be used, allowing researchers to identify bogus data submissions.

**Fig 2:**
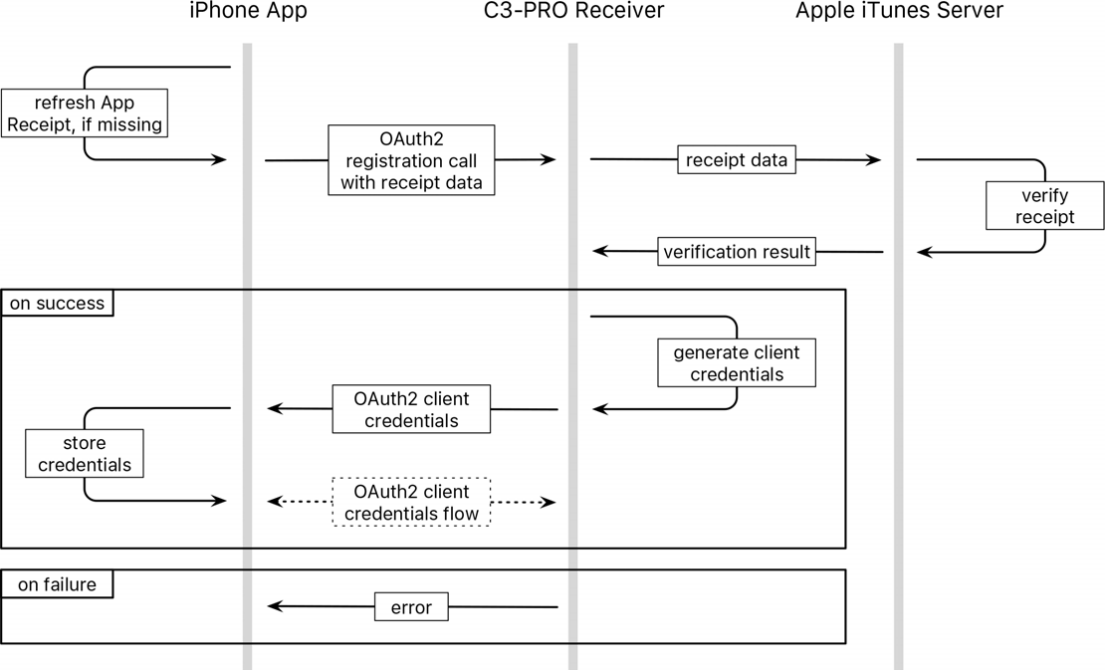
OAuth2 dynamic client registration flow, extended to use the app's App Store receipt. The receipt data is sent alongside the standard OAuth2 registration parameters and verified with Apple's iTunes servers. If the receipt is valid, standard dynamic client registration continues. Subsequently the app can request access tokens with the supplied client key and -secret through an OAuth2 "client credentials” flow.

To verify that data sent to our backend was indeed collected by a legitimate installation of a C3-PRO app we implemented a 2-legged OAuth2 "client credentials" authorization flow [11] using Apple's App Store receipt. This receipt is a PKCS #7 envelope uniquely assigned to an app version, device and device-user combination, issued and signed by Apple which can be verified with Apple's servers. To receive client credentials, a new install of the app sends its receipt to our receiver server, which verifies it against Apple's servers and returns a client key and client secret to the app, if verification succeeds. These credentials are subsequently stored in the device keychain and can be traded for an access token when data needs to be sent to the receiver (Fig 2).

### Data Format

The conversion between ResearchKit's in-memory representation and FHIR resources is handled by our iOS framework. A FHIR "Contract" resource contains eligibility criteria and informed consent material. Surveys are represented in a FHIR "Questionnaire", allowing creation of a repository of publicly available surveys, while responses are collected in FHIR "QuestionnaireResponse" resources. Appropriately, resources are linked via FHIR "Reference" properties, which allows association of PRO data with the respective trial participant. At the time of writing, the framework uses the DSTU-2 version (1.0.2) of FHIR from October 2015 [12]. C3-PRO will continually be updated to support the latest FHIR specifications.

### Consent and Privacy

After a participant has viewed all consenting material and agrees to participate, name and signature are captured. We create a "Patient" resource, which only contains the UUID, birth-year (if provided) and the first three digits of the zip code, to be compliant with HIPAA's Safe Harbor guidelines (*http://www.hhs.gov/ocr/privacy/hipaa/understanding/coveredentities/De-identification/guidance.html*), along with the US state abbreviation, and return it to our server. The zip code is obtained using reverse geocoding based on the device's current location at the time of enrollment and also used to determine the state. The FHIR "Contract" resource containing informed consent material then references the patient resource and is also returned to the research infrastructure in order to capture consent.

The participant's signature is stored as image data on device and used to generate a PDF file for personal use, containing name, date and signature. The participant can review the consent and share it via email or other means with herself or third parties at any time. Because the consent contains name and signature, it is not sent to the research backend.

### Data Submission

To avoid making a participant wait for data upload, our framework implements a first-in-first-out (FIFO) data queue. In case an upload fails, the respective resource is stored as a file in a local directory controlled by the queue. All files in the queue's directory receive the same name with an appended index number. When saving a resource, the directory is scanned for existing resources and a filename with the largest index number is created. Resubmission is periodically attempted with the FIFO queue ensuring the correct resource order based on the index number in the filename. This prevents situations such as study data being sent before the "Contract" resource-indicating participant consent-is received.

### Data Security

Before submission, FHIR resources are symmetrically encrypted using the Advanced Encryption Standard (AES) [13] and a randomly generated key of 256 bit length. The random key is asymmetrically encrypted using the RSA crypto-system [14] and a 2048 bit public key. The AES-encrypted resource and the RSA-encrypted random key are then sent to our receiver server in a straightforward JSON document containing four items: an id identifying the public key used for RSA encryption, the RSA-encrypted AES key, the AES-encrypted FHIR resource and the FHIR version number of the encrypted resource. This enables researchers to use public, non-trusted web hosting services such as AWS to host the receiver even for protected health information (PHI).

Resources stored in the device's FIFO queue receive the "complete-unless-open" data protection scheme provided by iOS. This designation uses hardware encryption: files protected as such are only readable while the user has unlocked the device [10].

### Web Traffic Filtering

In order to protect the research server from malicious web traffic, only the receiver server is publicly exposed. Incoming requests are first checked for the presence of a simple "Antispam" token in the request header, a random string embedded in the app binary known to the receiver component.

**Fig 3:**
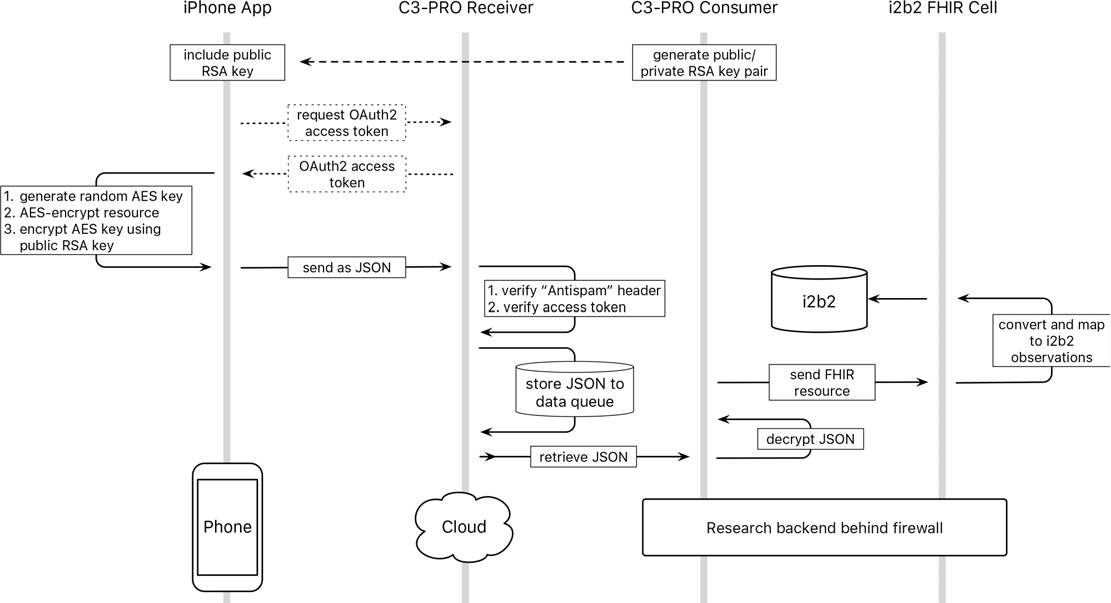
C3-PRO data flow. Data flow from data capture on the phone through the C3-PRO receiver, consumer and i2b2 cell into the i2b2 database.

As a second step the actual "Authorization" header is inspected for a valid OAuth2 access token. This token is handed out to the app after a "client credentials" OAuth2 flow, using client key and secret handed out to the app after registration described above (Fig 3).

Resources submitted with a valid access token are made available to the C3-PRO consumer, which is securely hosted on servers in-house. The consumer continuously receives encrypted FHIR resources along with the encrypted randomly generated AES keys and, using the corresponding private key, decrypts the AES key which it uses to decrypt the FHIR resource bodies. These FHIR resources are then forwarded to our i2b2 FHIR cell.

### Storage

Our FHIR compliant i2b2 cell receives incoming data and converts the FHIR resources to i2b2 observations. Depending on resource type, the resource is 1) imported into the i2b2 instance or 2) stored in a separate schema.

1. Resources of type "QuestionnaireResponse", "Observation" and "Patient" are imported into our i2b2 instance. In case of "QuestionnaireResponse" and "Observation" resources, the import pipeline generates one i2b2 observation for each answer or activity data point in an event timestamped with the resource's specified date and time. Multiple choice answers are de-normalized by creating one observation per choice. In case of the "Patient" resource, the i2b2 patient dimension is updated with the US state abbreviation.
2. "Contract" resources, indicating participant consent, are stored in a separate schema. Since incoming participant data is already de-identified when it arrives at the i2b2 instance, the UUID serves as link between consent and study data.

Storing survey responses as individual observations greatly facilitates future data analysis and enables proper utilization of already existing i2b2 plug-ins. For example, the question in the C Tracker survey, “Which hepatitis C antiviral medications are you on?” allows the participant to select one or more medications on the iPhone screen. Associated with each choice are RxNorm concept identifiers (RxCUI). Responses then contain the RxCUI of chosen drugs, allowing i2b2 to store these drugs alongside drugs from other sources, such as a hospital electronic medical record systems, greatly simplifying data evaluation. Additionally, i2b2 data is accessible not only through direct interaction with its database but also through an ontology that recreates the different surveys. The ontology is established at the time of survey creation to ensure a comprehensive posterior analysis.

## Discussion

We have built a complete, open source toolchain connecting ResearchKit to the widely used i2b2 research infrastructure, leveraging the emerging FHIR standard, with the aim to further commoditize creation of iPhone research apps. The toolchain was developed alongside our “C Tracker” app, a study collecting PRO and activity data from anonymous hepatitis C patients [6,7]. By sharing code on the widely used GitHub (*https://github.com*) platform and setting up a Google Group (*https://groups.google.com#!forum/c3-pro-developers*) for help and discussion, we hope to improve and extend the toolchain with input from the community.

In this first iteration we have focused on “in the wild” recruitment, where participants stay anonymous both for their comfort and protection, only identifiable by a randomly generated key. Data collected on devices is encrypted using public-key cryptography, hence an app does not embed a compromisable secret. Encrypted, PHI data can be securely transmitted via publicly available hosting services, which are professionally managed and able to handle a wide spectrum of web traffic. Only authenticated submissions are forwarded to researcher-owned i2b2 instances for storage and evaluation, cutting the risk of a targeted attack. Importantly, with deployments at over 120 medical centers, i2b2 is well known to biomedical researchers and health systems, greatly facilitating extension of research studies with data collected through C3-PRO.

For convenience, our iOS framework removes the need to programmatically create and evaluate surveys by using FHIR Questionnaire and QuestionnaireResponse resources, respectively. These file-based formats can both be obtained and reported back to the researcher's web server via a standardized RESTful service interface. Sensory data capturing user activity is encoded in the same file format, allowing storage and evaluation of such data in the same manner as survey data.

By randomly generating–but permanently storing–a patient identifier, in combination with verifying app installs with the app's App Store receipt, there is no need for the participant to create an account nor to log in. We have purposefully chosen this approach for participants in our C Tracker study, which enrolls hepatitis C patients who may not be comfortable with divulging their name or email address due to stigma associated with the infection [15].

Our “account-less” approach does however not allow a participant to resume enrollment on a different device. The UUID is included in encrypted iOS device backups, hence setting up a new device from backup keeps UUID and enrollment intact. However, should a participant manually re-download the research app to her new device, existing enrollment is not detected and the user must restart trial involvement by re-enrolling as a new participant. This issue can potentially be addressed by an out-of-band mechanism with export and subsequent import of the UUID by the research app, allowing the subject to resume trial participation “identified” as the original participant.

Lastly, while C3-PRO is targeted at the international research community, our first implementation of C3-PRO (C Tracker) primarily accommodates US laws. National laws governing human subjects research may require further technological advances, especially regarding electronic informed consent.

## Conclusions

C3-PRO is a secure, end-to-end solution for researchers wanting to utilize ResearchKit in combination with an i2b2 research backend to enable direct patient enrollment into clinical trials. We expect that such toolchains further lower the barrier for smartphone research app creation, enabling more research groups to take advantage of PRO capture via smartphone apps.

Work to enable data-linkage with already known participants, for example participants in an existing cohort, is underway. Furthermore, an adaptation to ResearchStack is planned, which will enable iPhone and Android research apps to be developed side-by-side. By relying on FHIR formats, we enable survey question and consent libraries to become standardized and used across studies.
